# HEALTH CONDITIONS, CONTROL BELIEFS, AND ATTITUDINAL BELIEFS OF USING TECHNOLOGY IN LOW-INCOME OLDER ADULTS

**DOI:** 10.1093/geroni/igad104.2206

**Published:** 2023-12-21

**Authors:** Eunice Oladepe Ojo, Ladda Thiamwong, Rui Xie, Nichole Lighthall, Joon-Hyuk Park, Victoria Loerzel, Jeffrey Stout

**Affiliations:** University of Central Florida, Orlando, Florida, United States; University of Central Florida, Orlando, Florida, United States; University of Central Florida, Orlando, Florida, United States; University of Central Florida, Orlando, Florida, United States; University of Central Florida, Orlando, Florida, United States; University of Central Florida, Orlando, Florida, United States; University of Central Florida, Orlando, Florida, United States

## Abstract

Integrating assistive technologies into fall prevention interventions could improve health outcomes, but older adults in low-income settings face greater challenges with technology use due to factors such as cost, access, and location. We examined the acceptance of technology in low-income older adults and the factors associated with their acceptance. In this cross-sectional study, 126 adults aged 60 years and older agreed to participate in the study. Three technologies, including a BTrack balance system (BBS), bioimpedance measurement device (InBody), and activity monitoring device (Actigraphs) were used to assess fall risk. The Senior Technology Acceptance (STA) was used to assess the acceptance of technology. STA consists of four domains with 14 items (1-10 rating scale). The Pearson correlation test was used to determine the correlation between STA scores, fall risk, and demographic factors. We found a significant correlation between STA and the CDC’s STEADI fall risk score (r = 0.3, p = 0.00075); between STA and education (r = 0.26, p = 0.0042); and between STA and age (r = -0.32, p = 0.00031). Participants had the highest mean STA scores to the lowest mean scores as follows: health conditions (41.9 ± 5.8), control beliefs (30.1 ± 8.1), attitudinal beliefs (20.9 ± 8.2), and gerontological anxiety (8.3 ± 5.7). The use of technology is acceptable to low-income older adults but fall risk, education, and age were associated with acceptance. They may benefit from technologically based fall prevention intervention since they have the potential to use technology with low fear expressed toward it.

